# Why hospital-based healthcare professionals do not report adverse drug reactions: a mixed methods study using the Theoretical Domains Framework

**DOI:** 10.1007/s00228-022-03326-x

**Published:** 2022-04-27

**Authors:** Raymond Li, Kate Curtis, Connie Van, Syed Tabish Razi Zaidi, Chin Yen Yeo, Christina Arun Kali, Mithila Zaheen, Grace Therese Moujalli, Ronald Castelino

**Affiliations:** 1 Faculty of Medicine and Health, University of Sydney, Parramatta Rd, Camperdown NSW 2006, Sydney, Australia; 2 Faculty of Medicine and Health, University of Leeds, Leeds, England; 3 Western Sydney Local Health District, Blacktown and Mt Druitt Hospital, Mt Druitt, Australia

**Keywords:** Pharmacovigilance, Adverse drug reactions, Reporting, Theoretical domains framework

## Abstract

**Purpose:**

Adverse drug reaction (ADR) underreporting is highly prevalent across the world. This study aimed to identify factors associated with ADR reporting and map these to a behavioural change framework to help inform future interventions designed to improve ADR underreporting.

**Methods:**

A mixed methods survey was distributed to healthcare professionals at a tertiary hospital in Sydney, Australia. Quantitative data was analysed using logistic regression to identify factors that predict ADR reporting. Qualitative data was evaluated using content analysis. These were then integrated and mapped to the 14 domains within the Theoretical Domains Framework (TDF) to identify target areas relevant for improving ADR reporting.

**Results:**

One hundred thirty-three healthcare professionals completed the survey. Knowing how to report ADRs (OR 4.56, 95%CI 1.95–10.7), having been trained on ADR reporting (OR 2.72, 95%CI 1.29–5.77), and encountering ADRs as part of clinical practice (OR 10.3, 95%CI 3.59–29.4) were significant predictors of reporting an ADR. Content analysis identified three categories: modifying the ADR reporting process, enabling clinicians to report ADRs, and creating a positive ADR reporting culture. After data integration, the three target TDF domains were knowledge, environmental context/resources, and beliefs about consequences.

**Conclusion:**

Future interventions designed to improve ADR reporting should address these target domains to instigate behaviour change in healthcare professionals’ reporting of ADRs.

**Supplementary information:**

The online version contains supplementary material available at 10.1007/s00228-022-03326-x.

## Introduction

Adverse drug reactions (ADR) are defined as any untoward medical occurrence in a patient administered a pharmaceutical product where a causal relationship is suspected [1]. They are a major cause of morbidity and mortality and directly responsible for up to 18% of hospital admissions and 27% of deaths in Australia [2]. The costs of ADRs are considerable due to the complexities associated with ADR treatment in a patient group who are generally older and taking more medications [3, 4]. The rate of ADR-related hospitalizations in Australia has increased by 21% from 8.0 per 100 hospitalizations in 2007/08 to 9.7 per 100 hospitalizations in 2015/16. Up to 60% of these were considered preventable [5].

Some ADR-related hospitalizations may be related to the poor characterization of the safety profiles of medicines due to underreporting of ADRs once a medicine is marketed [6]. In addition, most ADR reports are of very low quality, with missing information that is required to make an informed assessment of the frequency, severity, and causal relationship between the ADR and medicine [6–8]. This causes delays for regulatory agencies to remove medicines with unacceptable safety profiles. A 2016 systematic review reported the median time taken to withdraw a medicine for safety reasons was 10 years after its launch [9].

Barriers to ADR reporting by clinicians include lack of time, competing clinical priorities, uncertainty about the causal relationship between the drug and ADR, difficulties in accessing the reporting form, length of reporting form, lack of a user-friendly electronic ADR reporting platform, lack of awareness, and a belief that all serious reactions are well documented by the time a medicine is marketed [10–14]. In addition, ADRs are diagnosed over time and may require the input of multiple healthcare professionals, while most ADR reporting forms only allow for reporting of ADRs at a specific time point [15]. Interventions to improve ADR reporting have not been designed to specifically address the known barriers. A 2020 systematic review of interventions to improve ADR reporting concluded that their effectiveness was modest and that there was a lack of consideration of theoretical frameworks in the design of interventions [16, 17]. In addition, end-user input from healthcare professionals into the design of ADR reporting systems is lacking with only the needs of regulatory agencies taken into account [18]. As such, a knowledge gap exists in the creation of an intervention that is designed specifically to address the key determinants of behaviour change required to improve the quantity and quality of ADR reporting.

This study investigated medical officer, nurse, and pharmacist perspectives of ADR reporting in a hospital setting, so target areas can be identified to inform the development of a tailored intervention to improve ADR reporting.

## Methods

### Study design

This was an embedded mixed methods study where a qualitative component was added to a primarily quantitative study and the data were collected and analysed together [19]. We conducted a cross-sectional survey of medical officers, nurses, and pharmacists practising at Blacktown Hospital, a tertiary referral hospital with 570 beds in Western Sydney [20]. Currently, healthcare professionals in this hospital report ADRs by completing a ‘blue card’ reporting form and submitting it to the Australian regulator, the Therapeutic Goods Administration (TGA), through an online portal, or by email, fax, or post [21]. However, other aspects of patient care in this hospital have migrated to electronic platforms such as eMedical Records and eMedication Management.

Ethics was obtained from the Western Sydney Local Health District (HREC reference: 2020/ETH00597).

### Survey development

We were interested in 3 specific areas, namely, knowledge, perspectives, and practices of ADR reporting based on a previous survey conducted for community pharmacists practising in Australia [22]. In addition, development of the survey was also guided by the Theoretical Domains Framework (TDF). The TDF was developed and validated by an international collaboration of behavioural scientists and implementation researchers to identify key factors that would influence behaviour change among healthcare professionals [23]. This led to the establishment of 14 domain areas including knowledge; skills; social/professional role and identity; beliefs about capabilities; optimism; beliefs about consequences; reinforcement; intentions; goals; memory, attention and decision processes; environmental context and resources; social influences; emotion; and behavioural regulation, of which any one or combination of these domains may be needed to cause behaviour change. The TDF has been used extensively in healthcare research in Australia to identify barriers that need to be addressed to increase uptake of a new process or system. Examples include the successful adoption of an electronic medicine management system in hospitals, implementing a blunt chest injury care bundle, and adopting guidelines for the management of acute low back pain [24–26]. The TDF was selected as it identifies a wide range of determinants of behaviour and can be mapped to the behaviour change wheel to identify suitable behaviour change techniques (BCT) to inform interventions for improving ADR reporting (Supplementary Index, Figure S1) [24].

A pool of questions for each of these areas was generated based on each of the 14 domains within the TDF, with the question selection based on consultations with senior clinicians from the investigators’ network with an interest in this topic and practising in hospital nursing, pharmacy, and medicine.

We created a draft survey tool containing questions answered on a 5-point Likert scale from strongly disagree to strongly agree and open-ended questions to collect additional information about ADR reporting. This was piloted with a group of hospital pharmacists and nurses (*n* = 5) for feedback, and the wording of two questions was revised to enhance their clarity to the audience. The final version of the survey tool contained 25 items and is shown in Appendix S1 (Supplementary index).

### Data collection and recruitment

All medical officers, nurses, and pharmacists at Blacktown hospital were emailed an invitation to participate in this study from their departmental director/manager. This email correspondence contained the participant information sheet and an electronic link to the survey. Posters with QR codes were circulated around the hospital inviting participants to complete the survey. Data were captured using the Research Electronic Data Capture (REDCap), which is a secure web-based database application maintained by the University of Sydney.

### Statistical analyses

Statistical analyses were conducted using IBM SPSS (version 27.0) with significance levels set at *P* < 0.05. Adjustment for multiple comparisons was made using the Bonferroni correction. Descriptive statistics were reported using medians and interquartile range (IQR) as the Shapiro–Wilk and Kolmogorov–Smirnov tests displayed significance indicating the data is not normally distributed. The Kruskal–Wallis test was used for comparing the perspectives towards ADR reporting between pharmacists, medical officers, and nurses, as well as those who reported ADRs versus those who didn’t. Multivariate logistic regression was used to identify factors that predict whether a healthcare professional reports an ADR.

Qualitative data from free text responses were analysed using the conventional content analysis approach to identify new themes and categories without bias towards pre-existing theories or frameworks on this topic [27]. Initially, the researchers familiarized themselves with the data by repeatedly reading through the entire content to achieve immersion and obtain an overall meaning. The data were then carefully analysed by searching for terminology that may capture a key thought or concept (sub-categories). Notes were made for each of these concepts, and labels were then assigned to help classify these into categories. This entire process was conducted by 3 researchers (RL, KC, and CV) independently. If there were discrepancies, the researchers discussed these, and a consensus approach was taken.

The quantitative and qualitative data were then integrated and mapped to each of the 14 TDF domains using a consensus approach by 3 investigators (RL, KC, and CV). Quantitative results that had a median score of 5 or greater (equivalent to strongly agree on 5-point Likert scale) were included in this integration phase. The domains were then classified as target domains if 3 or more quantitative or qualitative results were mapped to that domain. This helped to identify the most important domains to target when designing future interventions to improve ADR reporting.

## Results

The survey was completed by 133 healthcare professionals comprised of 16 pharmacists (12.0%), 76 nurses (57.1%), and 41 medical officers (30.8%). Most respondents had encountered ADRs in their clinical practice (66.4%) indicating that they have either treated an ADR for a patient or a patient reported an ADR to them in a consultation. However, less than half of these healthcare professionals have reported an ADR (41.8%). Over one third of healthcare professionals did not know how to report ADRs to the hospital safety committee (34.3%) or the TGA (35.1%). Almost two thirds of healthcare professionals (64.9%) indicated they had not received any training on ADR reporting. The vast majority (94%) were not aware of the recently introduced TGA black triangle scheme, and very few were subscribed to receive TGA safety alerts (15.7%).

### Quantitative: differences among pharmacists, medical officers, and nurses

Most healthcare professionals agreed that ADR reporting is important for patient care (94.7%) and that they have a professional obligation to report ADRs (94.0%). Pharmacists and nurses reported better knowledge on how to report ADRs to the hospital safety committee as well as the TGA than medical officers (reporting to hospital committee, 75% and 76.3% vs 41.5%, *P* < 0.001, and reporting to TGA, 100% and 72.4% vs 36.6%, *p* < 0.001). Furthermore, more pharmacists reported they had received training on ADR reporting than medical officers (43.7% vs 22.0%, *P* = 0.013). Three quarters of pharmacists have reported an ADR, and this was substantially more than medical officers (46.3%) and nurses (31.6%) even though there were no differences among the three healthcare professional groups in encountering ADRs in their clinical practice (Table 1). Multivariate logistic regression showed that knowing how to report ADRs to the hospital committee (OR 4.56, 95%CI 1.95–10.7), having been trained on ADR reporting (OR 2.72, 95%CI 1.29–5.77), and encountering ADRs as part of clinical practice (OR 10.3, 95%CI 3.59–29.4) were significant predictors of making an ADR report (Table 2).

**Table 1:** Characteristics of respondents by healthcare professional type

	**Medical officer**	**Nurse**	**Pharmacist**	**Overall**	***P* value**
***N* (%)**	41 (30.8)	76 (57.1)	16 (12.0)	133	
**Median no. of years of clinical practice (IQR)**	3.0 (1.0–5.5)	8.0 (3.0–14.75)	5.0 (1.5–10.75)	5 (2–12)	
**Median no. hours per week (IQR)**	40 (39–42.5)	40 (38–40)	40 (38–40)	40 (38–40)	
**Qualification**					
Undergraduate (%)	39.0	50.0	43.8	45.9	
Postgraduate (%)	61.0	50.0	56.3	54.1	
**Know how to report ADRs to hospital safety committee**					
Yes (%)	41.5	76.3	75.0	65.4	< 0.001^a^
No (%)	58.5	23.7	25.0	34.6	< 0.001^b^
**Know how to report ADRs to TGA**					
Yes (%)	36.6	72.4	100	64.7	< 0.001^a^
No (%)	63.4	27.6	0.0	35.3	< 0.001^b^
**Received training on ADR reporting**					
Yes (%)	22.0	36.8	43.7	34.6	0.013^a^
No (%)	78.0	63.2	56.3	65.4	
**Aware of black triangle scheme in Australia**					
Yes (%)	2.4	3.9	18.8	5.3	n.s
No (%)	97.6	96.1	81.2	94.7	
**Subscribed to receive TGA safety alerts**					
Yes (%)	7.3	17.1	25.0	15.0	n.s
No (%)	92.7	82.9	75.0	85.0	
**Encountered ADR in clinical practice**					
Yes (%)	73.2	59.2	81.3	66.2	n.s
No (%)	26.8	40.8	18.8	33.8	
**Have reported ADR**					
Yes (%)	46.3	31.6	75.0	41.4	0.001^c^
No (%)	53.7	68.4	25.0	58.6	

Significance value was set at 0.017 after applying the Bonferroni correction

*n.s.* not significant

^a^Medical officer vs pharmacist

^b^Medical officer vs nurse

^c^Nurse vs pharmacist

**Table 2:** Factors associated with healthcare professional reporting of ADR

**Factor**	**Absolute number (Y/N)**		**Crude odds ratio (95% CI)**	**Adjusted odds ratio^ (95% CI)**	***P* value**
**Know how to report ADR to hospital safety committee**	**87**	**46**	**4.61 (1.99–10.7)**	**4.56 (1.95–10.7)**	** < 0.001**
Know how to report ADR to TGA	86	47	3.42 (1.55–7.61)	3.34 (1.49–7.46)	0.003
Received training on ADR reporting	46	87	2.60 (1.25–5.42)	2.72 (1.29–5.77)	0.009
Awareness of TGA black triangle scheme	7	126	9.43 (1.10–80.7)	9.25 (0.35–55.8)	0.25
Subscribed to receive TGA safety alerts	20	113	1.51 (0.58–3.92)	1.36 (0.51–3.67)	0.54
Encountered ADR in clinical practice	88	45	10.5 (3.79–29.2)	10.3 (3.59–29.4)	< 0.001

^results were adjusted for no. years of clinical practice, no. of hours per week, and highest qualification

### Quantitative: perspectives of healthcare professionals towards ADR reporting

Healthcare professionals agreed that ADR reporting should be made mandatory (median [IQR], 5 [3–5]) is important for patient care (median [IQR], 5 [4, 5]) and that they have a professional obligation to report ADRs (median [IQR], 5 [4, 5]) with no significant differences in these perspectives among physicians, nurses, or pharmacists. Healthcare professionals believed they were more likely to report ADRs if there was an electronic tool that automatically populates information from existing datasets (median [IQR], 5 [4, 5]). The median scores for the perspectives of healthcare professionals towards ADR reporting are presented in Table 3.

**Table 3:** Perspectives of medical officers, nurse, and pharmacists towards ADR reporting (1–5 Likert scale)

**Question (TDF domain)**	**Median (IQR), overall**	**Median (IQR), medical officer**	**Median (IQR), nurse**	**Median (IQR), pharmacist**
Reporting ADRs is important for patient care (beliefs about consequences)	5 (4–5)	4 (4–5)	5 (4–5)	5 (4–5)
Reporting ADRs should be mandatory for HCPs (behavioural regulation)	5 (4–5)	4 (4–5)	5 (4–5)	4.5 (4–5)
I have a professional obligation to report ADRs (social/professional role and identity)	5 (4–5)	4 (4–5)	5 (4–5)	5 (4–5)
The safety profile of medicines is well characterized by the time it is marketed (knowledge)	4 (3–4)	3 (3–4)	4 (3–5)	3 (2–4)
I’m interested in reading about ADRs in medical literature (reinforcement)	4 (3–5)	4 (3–4)	4 (3–5)	4.5 (4–5)
**I’m more likely to report ADRs if:**				
There was an incentive (reinforcement)	3 (3–4)	3 (2–4)	3 (3–4)	3 (2–4)
There was an electronic tool that automatically populates information from existing datasets (environment context and resources)	5 (4–5)	4 (4–5)	5 (4–5)	5 (4–5)
I’m mandated to report and there is a consequence if I don’t (beliefs about consequences)	4 (3–4)	4 (3–4.5)	4 (3–4)	4 (3–4.75)
There was a hospital protocol mandating ADR reporting (behavioural regulation)	4 (3–5)	4 (3–4)	4 (3–4.75)	4 (4–5)
I see that there are other HCPs reporting ADRs (social influences)	4 (4–5)	4 (4–4)	4 (3–5)	4 (3–5)
There was a reminder (environment context and resources)	4 (3–4)	4 (3–4)	4 (3–4)	4 (2.25–5)
It was serious and unexpected (beliefs about consequences)	5 (4–5)	5 (4–5)	4 (4–5)	5 (4–5)
It was for a new medicine (intention)	4 (3–5)	4 (4–5)	4 (3–5)	5 (4–5)
It has a strong causal association with the medicine (beliefs about consequences)	4 (3–5)	4 (4–5)	4 (3–5)	4 (3.25–5)
There is someone monitoring our ADR reporting (behavioural regulation)	4 (3–4.5)	4 (3–4)	4 (3–4)	4 (3.25–5)
I receive an acknowledgement (reinforcement)	4 (3–4)	4 (3–4)	4 (3–4)	3 (3–4)
**I’m less likely to report ADRs because:**				
I don’t have the time (environment context and resources)	3 (2–4)	4 (2.5–4)	3 (2–4)	4 (2.25–4)
I fear there may be legal repercussions (beliefs about consequences)	2 (2–3)	2 (2–3)	3 (2–3)	2 (1–2)
There are no results or actions taken based on ADRs I report (beliefs about consequences)	3 (2–4)	3 (2–4)	3 (2–4)	3 (2–4)
I forget to report at the time (memory, attention, and decision processes)	3 (2–4)	3 (2–4)	3 (2–4)	4 (3–4)
It was non-serious and expected (knowledge)	3 (2–4)	4 (3–4)	3 (2–4)	4 (3–4)
I don’t have enough information to warrant a report (environment context and resources)	3 (3–4)	3 (3–4)	3 (2–4)	4 (2.25–4)
I don’t know how to report (skills)	3 (2–4)	4 (3–4)	3 (2–4)	2 (2–3)
I’m uncertain of the causal relationship (knowledge)	3 (3–4)	4 (3–4)	3 (2–4)	3 (2.25–4)
I would rather have it published in the medical literature (intentions)	3 (2–3)	3 (3–4)	3 (2–3)	2 (1–2.75)
I don’t know when I’m supposed to (knowledge)	3 (–4)	4 (3–4)	3 (2–4)	2 (1.25–3)
It won’t make a difference (optimism, beliefs about consequences)	3 (2–3)	3 (2–4)	3 (2–3)	2 (1–3.75)
My colleagues don’t (social influence)	3 (2–3)	3 (2–4)	3 (2–3)	2 (1–3.75)
It would cause stress and burnout in my workload (emotion)	3 (2–4)	3 (2–4)	3 (2–4)	2.5 (1.25–3.75)
I have been encouraged not to (social influence)	2 (1–3)	2 (1–3)	2 (2–3)	2 (1–2)

*IQR* interquartile range

### Qualitative: content analysis

There were 110 healthcare professionals who responded to the qualitative component of the survey. Their responses were classified into 245 sub-categories. These were then synthesized into 3 main categories: modifying the ADR reporting process; enabling clinicians to report ADRs, and creating a positive ADR reporting culture (Table 4).

**Table 4:** Categorization of qualitative data

**Category and *sub-categories (number of respondent comments and TDF domain)***
**Modifying ADR reporting process**
* Automate the reporting process (n* = *6)* (environment context and resources) * Make ADR reporting mandatory/through protocols (n* = *18) (behavioural regulation)* * Use electronic tools/software to assist ADR reporting (n* = *5)* (environment context and resources) * Lack of time to report ADRs (n* = *50)* (environment context and resources) * Forget to report ADRs (n* = *7) (memory, attention, and decision processes)* * Make ADR reporting easier (n* = *29) (environment context and resources)* * High workload/lack of resources (n* = *14)* (environment context and resources) * Creating reminders to assist ADR reporting (n* = *4)* (environment context and resources)
**Enabling clinicians to report ADRs**
* Provide education to drive knowledge and awareness of ADR reporting (n* = *62) (knowledge)* * Provide training on ADR reporting process (n* = *21) (knowledge)* * Uncertain of causal relationship to warrant an ADR report (n* = *10) (knowledge)* * Non-serious ADRs do not need to be reported (n* = *3) (knowledge)*
**Creating a positive ADR reporting culture**
* Providing acknowledgement/feedback for reported ADRs (n* = *3) (reinforcement)* * Incentivize the reporting of ADRs (n* = *4) (reinforcement)* * Fear of legal repercussions for reporting ADRs (n* = *2) (beliefs about consequences)* * Provide encouragement for colleagues to report ADRs (n* = *7) (social influences)*

#### Modifying the ADR reporting process

Modifying the ADR reporting process, particularly through streamlining, improving, and mandating ADR reporting by healthcare professionals, was the most identified category, represented by 133 comments. Of these, there were 29 comments specifically on ‘making the ADR reporting process easier’ and 18 comments on making ADR reporting mandatory through monitoring, protocols, or setting key performance indicators. There were 11 respondents that highlighted forgetfulness at the time of ADR occurrence as a barrier to reporting and that reminders would serve as an important intervention to assist with the reporting process:

Make the whole process easier, formulate a protocol, and screen databases using automation. (Medical officer, respondent ID 052).

However, even if mandated, there remains several barriers to this as there were 50 comments on a ‘lack of time and/or resources to report ADRs:

Lack of time due to workload – staff won’t have time for breaks. (Nurse, respondent ID 070).

#### Enabling clinicians to report ADRs

Enabling clinicians to report ADRs by increasing their knowledge, awareness, and understanding of both the importance and process of ADR reporting was highlighted by 96 respondent comments. There were 62 comments that highlighted the need to provide education around ADR knowledge and awareness while 21 comments emphasized the importance of offering training sessions on the ADR reporting process. Thirteen comments indicated any ‘uncertainty of a causal relationship between the ADR and the medicine’ and ‘non-serious reports’ would be barriers to submitting an ADR report:

Being unsure of the causal relationship between the reaction and the drug, as well as often needing to confer with other healthcare professionals about whether or not they would consider something to be an adverse drug reaction or a natural progression of a patient’s condition. (Pharmacist, respondent ID 003).

#### Creating a positive ADR reporting culture

Creating a positive ADR reporting culture was highlighted by 16 comments as being important to facilitate ADR reporting. These include providing encouragement for colleagues to report ADRs (*n* = 7), providing acknowledgement and/or feedback for reported ADRs (*n* = 3), and incentivizing the reporting of ADRs (*n* = 4). Fear of legal repercussions was identified as a barrier to creating that positive ADR reporting culture (*n* = 2):

Reporting culture will definitely help increase rates of ADR reporting, this will need to be facilitated/encouraged/embedded by senior clinicians/managers/executives. (Pharmacist, respondent ID 001).

### Integration: mapping of quantitative and qualitative results into domains within the TDF

Quantitative items with scores > 4 and categorized qualitative results were mapped to 9 TDF domains: knowledge; social/professional role and identity; beliefs about consequences; reinforcement; intentions; memory, attention and decision processes; environment context and resources; social influences; and behavioural regulation. From this, the 3 domains selected as targets for future interventions to improve ADR reporting were determined to be knowledge [7 results]; environment context and resources (7 results); and beliefs about consequences (4 results) — see Table 5.

**Table 5:** Quantitative and qualitative results mapped to the TDF to identify target domains that inform future interventions to improve ADR reporting

**Domain (description)**	**Quantitative result(s)**	**Qualitative result(s)**	**Target domain (Y/N)**
**1. Knowledge** (an awareness of the existence of something)	65.7% know how to report ADRs to the hospital safety committee 64.9% know how to report ADRs to the TGA 35.1% have received training on ADR reporting	**Facilitators:** Providing education to drive knowledge/awareness of ADR reporting Provide training on ADR reporting process **Barriers:** Uncertain of causal relationship to warrant ADR report Non-serious ADRs do not need to be reported	Y
**2. Skills** (an ability or proficiency acquired through practice)			N
**3. Social/professional role and identity** (a coherent set of behaviours and displayed personal qualities of an individual in a social or work setting)	I have a professional obligation to report ADRs (median score 5 out of 5)		N
**4. Belief about capabilities** (acceptance of the truth, reality, or validity about an ability, talent, or facility that a person can put to constructive use)			N
**5. Optimism** (the confidence that things will happen for the best or that desired goals will be attained)			N
**6. Beliefs about consequences** (acceptance of the truth, reality, or validity about outcomes of a behaviour in a given situation)	More likely to report if the ADR was serious and unexpected (median score 5 out of 5) Reporting ADRs is important for patient care (median score 5 out of 5)	**Barriers:** Fear of legal repercussions for reporting ADRs	Y
**7. Reinforcement** (increasing the probability of a response by arranging a dependent relationship, or contingency, between the response and a given stimulus)		**Facilitators:** Providing acknowledgement/feedback for reported ADRs Incentivize the reporting of ADRs	N
**8. Intentions** (a conscious decision to perform a behaviour or a resolve to act in a certain way)			N
**9. Goals** (mental representations of outcomes or end states that an individual wants to achieve)			N
**10. Memory, attention, and decision processes** (the ability to retain information, focus selectively on aspects of the environment, and choose between two or more alternatives)		**Barriers:** Forget to report ADRs	N
**11. Environment context and resources** (any circumstance of a person’s situation or environment that discourages or encourages the development of skills and abilities, independence, social competence, and adaptive behaviour)	More likely to report if there was an electronic tool that automates ADR reporting (median score 5 out of 5)	**Facilitators:** Automate the reporting process Use electronic tools/software to assist ADR reporting Creating reminders to assist ADR reporting **Barriers:** Lack of time to report ADRs Make ADR reporting easier High workload/lack of resources	Y
**12. Social influences** (those interpersonal processes that can cause individuals to change their thoughts, feelings, or behaviours)		**Facilitators:** Provide encouragement for colleagues to report ADRs	N
**13. Emotion** (a complex reaction pattern, involving experiential, behavioural, and physiological elements, by which the individual attempts to deal with a personally significant matter or event)			N
**14. Behavioural regulation** Anything aimed at managing or changing objectively observed or measured actions)	Reporting ADRs should be mandatory for HCPs (median score 5 out of 5)	**Facilitators:** Make ADR reporting mandatory through protocols	N

## Discussion

This study identified several influences on clinician behaviour that need to be addressed in any future intervention designed to improve ADR reporting, in particular, knowledge, the work environment/resources, and beliefs about consequences.

We have applied the first two steps of French et al.’s [28] four-step model for change; the identification of (1) what and who needs to change, (2) what barriers and facilitators need to be addressed, (3) what interventions could be used to overcome the barriers, and (4) the evaluation of any intervention [28]. This study identified that (1) staff and systems within the workplace need to change and (2) barriers to be addressed include knowledge, environment, reinforcement, and memory. The next step would be to create a multifaceted intervention designed to overcome these barriers utilizing education, training, and environmental restructuring [29].

Knowledge was a key gap identified as over one third of respondents did not know how to report ADRs to either their hospital safety committee or the Australian regulator, while sub-categories collected from qualitative comments include the need to provide education and training on the importance of ADR reporting and process. This is consistent with previous studies which showed that a significant number of healthcare professionals were not educated or trained on ADR reporting, impacting their ability to report ADRs in their clinical practice [11, 22]. Consideration needs to be given in including ADR reporting into the curriculums of university healthcare degrees as well as part of continuing education workshops for healthcare professionals. Furthermore, training on ADR reporting can be included as part of the onboarding process for new healthcare professionals employed in a hospital setting.

Environment context and resources was also influential in healthcare professionals reporting of ADRs. Most healthcare professionals agreed that they would be more likely to report ADRs if the process was amended by adopting an electronic tool that is capable of automation. In addition; ‘modifying the ADR reporting process’ through making reporting mandatory, automating the reporting process, and utilizing electronic tools/software was proposed to facilitate ADR reporting. This is consistent with other literature which showed considerable interest among healthcare professionals towards uptake of new technologies to assist with ADR reporting [30]. A 2020 systematic review also showed that electronic strategies were more successful at improving ADR reporting rates than traditional interventions such as providing education and training [16]. However, it is also important to note that mandatory ADR reporting has not been shown to significantly improve ADR reporting in jurisdictions that have adopted this as it causes an excessive burden [31]. Therefore, any future interventions designed to improve ADR reporting should be developed around a digital framework to simplify and automate the process.

Lack of time, resources, and high workload were also identified as significant barriers to ADR reporting. This was expected as healthcare professionals are more likely to focus on treating the ADR at the time of occurrence, rather than thinking about reporting it. This is reinforced by staff indicating they are more likely to report ADRs if a reminder was created within the ADR reporting pathway. These results are also consistent with previous studies which showed that lack of time and resources are key issues that need to be overcome to encourage ADR reporting [32–36]. In addition, qualitative studies in Canada showed that reporting required duplication of documentation resulting in time constraints, and many ADR reporting systems were too complex and poorly fitted into clinical practice [14, 15]. Therefore, simplification of the reporting process so that ADR reporting is not perceived as an administrative burden is an essential consideration when designing future interventions.

Beliefs about consequences were the final target domain assessed as relevant to inform future interventions to improve ADR reporting. The quantitative results clearly showed that healthcare professionals were more likely to report ADRs if it was serious and unexpected or if there was a strong causal relationship between the medicine and the ADR. This may be due to the perception that regulators are more likely to take action and their report will be of consequence to characterizing the safety profile of the suspect medicine. In addition, a very strong perception that ADR reporting is important for patient care was identified. This was reinforced by the finding that HCPs in our study felt a very strong professional obligation to report ADRs. However, there were a couple of respondents who noted in the qualitative comments that fear of legal repercussions was a barrier for them to report ADRs despite a very neutral effect when this question was asked in the quantitative component of the survey.

The three TDF domains identified in this study were also identified in a 2015 Iranian study by Mirbaha et al. involving hospital pharmacists and nurses [10]. In that study, respondents admitted that they had low awareness on what ADRs should be reported (poor knowledge) and that special education and training should be provided on what and how ADRs should be reported. Within the domain of environment context and resources, the authors identified lack of time, complicated administrative procedures in the reporting process, and limited access to appropriate resources for submitting ADR reports, which were similar themes captured in our study. In the area of beliefs about consequences, comments around the importance of ADR reporting to enhance patient care and quality use of medicines were identified, which was also similar to our study. Mirbaha et al. also mapped their results to 3 additional TDF domains which were not classified as target domains in our study; these were skills, intention, and social influences. These differences may be explained by the different hospital working environments and culture experienced by clinicians in the management of ADR reporting.

Future intervention at the study site to improve ADR reporting must adopt a multifaceted approach using mechanisms known to address the identified barriers [16, 37]. An electronic tool incorporating automation and integration with existing hospital electronic health records/medication management systems can be deployed to help simplify the ADR reporting process to save time and resources, addressing the needs within the domain of environment context and resources. A systematic review showed that digital reporting tools were moderately successful with a doubling in the quantity of ADR reports; however, these tools required promotion to healthcare professionals [38]. Therefore, educational sessions on the importance and process of ADR reporting should be combined with any training sessions on how to use the new ADR reporting system. This would enable clinicians to report ADRs and address the gaps within the knowledge domain. Finally, a focus should be placed on ADR reporting for reactions that are serious, unexpected, and/or has a strong causal relationship with the suspect medicine. This would assist regulators with identifying new safety issues for medicines and fully characterize their safety profile.

### Study limitations

One of the key limitations for this study was the limited sample size of 133 healthcare professionals at a single hospital, indicating that these results may not be representative of the perspectives of all clinicians. This also had an impact on the results of the logistic regression as shown by the relatively wide confidence intervals. The low rate of participation was mainly due to the challenges of lock-down due to surging COVID-19 cases in Australia at the time this survey was deployed. Secondly, respondents completing this survey may be subject to social desirability bias [39]. Some healthcare professionals may feel guilty for not reporting ADRs and therefore are not likely to admit this. In addition, the respondents may have provided ‘socially desirable’ responses about their perspectives towards ADR reporting resulting in inflated scores in this area. However, the use of anonymized surveys may have reduced the impact of this bias. Thirdly, this study focused its enquiry specifically on behaviour change, which limited the exploration of other important factors such as end-user involvement in the design of any future ADR reporting tool. This is critically important as the needs of frontline clinicians must be considered to ensure any future interventions are successful. A study undertaken to pilot an electronic health record-based ADR reporting form with pharmacists showed that this was a critically important step to help inform the design and enhance the functionality of these features prior to its full implementation [40].

## Conclusion

A substantial proportion of healthcare professionals do not report ADRs. By using behaviour change theory, the factors associated with ADR reporting were mapped to three target domains of knowledge, environment context and resources, and beliefs about consequences. A multifaceted intervention addressing these domains should be implemented to instigate behaviour change in healthcare professionals’ reporting of ADRs.

## Supplementary information

Below is the link to the electronic supplementary material.

Supplementary file1 (DOCX 553 KB)

## References

[1] Glossary of terms used in pharmacovigilance: Uppsala Monitoring Center (2018) updated 06 April 2018

[2] Runciman WB, Roughead EE, Semple SJ, Adams RJ (2003). Adverse drug events and medication errors in Australia. Int J Qual Health Care.

[3] Davies EC, Green CF, Taylor S, Williamson PR, Mottram DR, Pirmohamed M (2009). Adverse drug reactions in hospital in-patients: a prospective analysis of 3695 patient-episodes. PLoS ONE.

[4] Rydberg DM, Holm L, Engqvist I, Fryckstedt J, Lindh JD, Stiller CO (2016). Adverse drug reactions in a tertiary care emergency medicine ward - prevalence, preventability and reporting. PLoS ONE.

[5] Australian Institute of Health and Welfare - adverse events treated in hospital 2018 cited 2021. Available from: https://www.aihw.gov.au/reports/australias-health/australias-health-2018/contents/indicators-of-australias-health/adverse-events-treated-in-hospital. Accessed 20 Jan 2022

[6] Li R, Curtis K, Zaidi STR, Van C, Thomson A, Castelino R (2021) Prevalence, characteristics, and reporting of adverse drug reactions in an Australian hospital: a retrospective review of hospital admissions due to adverse drug reactions. Expert Opin Drug Saf 1–8 10.1080/14740338.2021.1938539 34077311

[7] Li R, Curtis K, Zaidi STR, Van C, Castelino R (2020). Effect of the black triangle scheme and its online educational campaign on the quantity and quality of adverse drug event reporting in Australia: a time series analysis. Expert Opin Drug Saf.

[8] Ribeiro A, Lima S, Zampieri ME, Peinado M, Figueras A (2017). Filling quality of the reports of adverse drug reactions received at the Pharmacovigilance Centre of Sao Paulo (Brazil): missing information hinders the analysis of suspected associations. Expert Opin Drug Saf.

[9] Onakpoya IJ, Heneghan CJ, Aronson JK (2016). Post-marketing withdrawal of 462 medicinal products because of adverse drug reactions: a systematic review of the world literature. BMC Med.

[10] Mirbaha F, Shalviri G, Yazdizadeh B, Gholami K, Majdzadeh R (2015). Perceived barriers to reporting adverse drug events in hospitals: a qualitative study using theoretical domains framework approach. Implement Sci.

[11] Nita Y, Batty KT, Plumridge RJ (2005). Adverse drug reaction reporting: attitudes of Australian hospital pharmacists and doctors. J Pharm Pract Res.

[12] Varallo FR, Guimaraes SD, Abjaude SA, Mastroianni PD (2014) Causes for the underreporting of adverse drug events by health professionals: a systematic review. Revista da Escola de Enfermagem da USP 48(4):739–47 10.1590/s0080-623420140000400023 25338257

[13] Herdeiro MT, Figueiras A, Polonia J, Gestal-Otero JJ (2005). Physicians' attitudes and adverse drug reaction reporting : a case-control study in Portugal. Drug Saf.

[14] Hohl C, Lexchin JR, Balka E (2015). Can reporting of adverse drug reactions create safer systems while improving health data?. CMAJ.

[15] Hohl CM, Small SS, Peddie D, Badke K, Bailey C, Balka E (2018). Why clinicians don't report adverse drug events: qualitative study. JMIR Public Health Surveill.

[16] Li R, Zaidi STR, Chen T, Castelino R (2020). Effectiveness of interventions to improve adverse drug reaction reporting by healthcare professionals over the last decade: a systematic review. Pharmacoepidemiol Drug Saf.

[17] Paudyal V, Al-Hamid A, Bowen M, Hadi MA, Hasan SS, Jalal Z (2020). Interventions to improve spontaneous adverse drug reaction reporting by healthcare professionals and patients: systematic review and meta-analysis. Expert Opin Drug Saf.

[18] Peddie D, Small SS, Badke K, Wickham ME, Bailey C, Chruscicki A (2016). Designing an adverse drug event reporting system to prevent unintentional reexposures to harmful drugs: study protocol for a multiple methods design. JMIR Res Protoc.

[19] Creswell JW, Plano Clark VL (2011) Designing and conducting mixed methods research. Oaks T, editor. CA: Sage

[20] Blacktown and Mount Druitt Hospital NSW Health Western Sydney Local Health District (2021) updated 18 Dec 2020; cited 2021. Available from: https://www.wslhd.health.nsw.gov.au/Blacktown-Mount-Druitt-Hospital/Blacktown-Mount-Druitt-Hospital. Accessed 20 Jan 2022

[21] Blue card adverse reaction reporting form: Australian Department of Health (2021) Available from: https://www.tga.gov.au/form/blue-card-adverse-reaction-reporting-form. Accessed 20 Jan 2022

[22] Li R, Curtain C, Bereznicki L, Zaidi STR (2018). Community pharmacists’ knowledge and perspectives of reporting adverse drug reactions in Australia: a cross-sectional survey. Int J Clin Pharm.

[23] Atkins L, Francis J, Islam R, O'Connor D, Patey A, Ivers N (2017). A guide to using the theoretical domains framework of behaviour change to investigate implementation problems. Implement Sci.

[24] Debono D, Taylor N, Lipworth W, Greenfield D, Travaglia J, Black D (2017). Applying the theoretical domains framework to identify barriers and targeted interventions to enhance nurses' use of electronic medication management systems in two Australian hospitals. Implement Sci.

[25] Kourouche S, Buckley T, Van C, Munroe B, Curtis K (2019). Designing strategies to implement a blunt chest injury care bundle using the behaviour change wheel: a multi-site mixed methods study. BMC Health Serv Res.

[26] Hall AM, Scurrey SR, Pike AE, Albury C, Richmond HL, Matthews J (2019). Physician-reported barriers to using evidence-based recommendations for low back pain in clinical practice: a systematic review and synthesis of qualitative studies using the theoretical domains framework. Implement Sci.

[27] Hsieh H-F, Shannon SE (2005). Three approaches to qualitative content analysis. Qual Health Res.

[28] French SD, Green SE, O'Connor DA, McKenzie JE, Francis JJ, Michie S et al (2012) Developing theory-informed behaviour change interventions to implement evidence into practice: a systematic approach using the theoretical domains framework. Implement Sci IS 7(1):38 10.1186/1748-5908-7-38 PMC3443064 22531013

[29] Michie S, Atkins L, West R (2014) The behaviour change wheel. London: Silverback Publishing

[30] De Vries ST, Wong L, Sutcliffe A, Houÿez F, Ruiz CL, Mol PGM et al (2017) Factors influencing the use of a mobile app for reporting adverse drug reactions and receiving safety information: a qualitative study. Drug Saf 40(5):443–55 10.1007/s40264-016-0494-x PMC5384960 28035492

[31] Gautron S, Wentzell J, Kanji S, Nguyen T, Kobewka DM, MacDonald E (2018). Characterization of serious adverse drug reactions in hospital to determine potential implications of mandatory reporting. Can J Hosp Pharm.

[32] AlShammari TM, Almoslem MJ (2018). Knowledge, attitudes & practices of healthcare professionals in hospitals towards the reporting of adverse drug reactions in Saudi Arabia: a multi-centre cross sectional study. Saudi Pharmaceutical Journal.

[33] Gahr M, Connemann B, Zeiss R, Schönfeldt-Lecuona C, Dreyhaupt J, Lazik C (2021) Reporting, handling, and subjective importance of adverse drug reactions among general practitioners: an exploratory cross-sectional survey. Expert Opin Drug Saf 1–7 10.1080/14740338.2021.1933430 34014779

[34] Hughes ML, Weiss M (2019). Adverse drug reaction reporting by community pharmacists—the barriers and facilitators. Pharmacoepidemiol Drug Saf.

[35] Lopez-Gonzalez E, Herdeiro MT, Figueiras A (2009). Determinants of under-reporting of adverse drug reactions: a systematic review. Drug Saf.

[36] Terblanche A, Meyer JC, Godman B, Summers RS (2017) Knowledge, attitudes and perspective on adverse drug reaction reporting in a public sector hospital in South Africa: baseline analysis. Hospital Pract (1995) 45(5):238–45 10.1080/21548331.2017.1381013 28914115

[37] Gonzalez-Gonzalez C, Lopez-Gonzalez E, Herdeiro MT, Figueiras A (2013). Strategies to improve adverse drug reaction reporting: a critical and systematic review. Drug Saf.

[38] Ribeiro-Vaz I, Silva AM, Costa Santos C, Cruz-Correia R (2016). How to promote adverse drug reaction reports using information systems a systematic review and meta-analysis. BMC Med Inform Decis Mak.

[39] Althubaiti A (2016). Information bias in health research: definition, pitfalls, and adjustment methods. J Multidiscip Healthc.

[40] Chruscicki A, Badke K, Peddie D, Small S, Balka E, Hohl CM (2016). Pilot-testing an adverse drug event reporting form prior to its implementation in an electronic health record. Springer Plus.

