# Supplementary material for: Why hospital-based healthcare professionals do not report adverse drug reactions: a mixed methods study using the Theoretical Domains Framework
Source: Eur J Clin Pharmacol. 2022 Apr 27;78(7):1165–75. doi: 10.1007/s00228-022-03326-x (PMC9043508; doi:10.1007/s00228-022-03326-x)

**Supplementary Index**

**Appendix S1 – Survey tool**

1. Pleases select your profession: (drop down)
2. Physician
3. Nurse
4. Pharmacist
5. How many years have you been registered to practice in Australia? (numerical dropdown)
6. What is your highest level of qualification? (drop down)
7. Undergraduate
8. Postgraduate
9. How many hours are you employed to work (on average) at the hospital per week? (numerical dropdown)

**Knowledge and skills**

1. Can you define an adverse drug reaction?
2. Yes
3. No
4. Does your hospital have a protocol or procedure for reporting adverse drug reactions?
5. Yes
6. No
7. Don’t know
8. I know how to report adverse drug reactions within the hospital
9. Yes
10. No
11. I know how to report adverse drug reactions to the Therapeutic Goods Administration
12. Yes
13. No
14. I have received training on how to report adverse drug reactions
15. Yes
16. No
17. I am aware of the black triangle scheme that was introduced to Australia in January 2018.
18. Yes
19. No
20. Are you subscribed to receive safety alerts from the Therapeutic Goods Administration?
21. Yes
22. No

**ADR reporting practices**

1. I have encountered adverse drug reactions in patients as part of my clinical practice
2. Yes
3. No
4. I have reported adverse drug reactions
5. Yes
6. No
7. If you were to report an ADR, who would you report it to? Tick all that apply (multiple tickbox)
8. Australian regulator – Therapeutic Goods Administration
9. Hospital drug safety committee
10. State and territory health network
11. Pharmaceutical manufacturer
12. Other:
13. Not sure
14. If you were to make an ADR report, what is the maximum time you would be willing to spend to complete and submit this report?
15. <1 minute
16. 1-5 minutes
17. 6-10 minutes
18. >10 minutes

**Perspectives (5 point Likert scale strongly disagree to strongly agree)**

1. Reporting adverse drug reactions is important for patient care
2. Reporting adverse drug reactions should be mandatory for healthcare professionals
3. I have a professional obligation to report adverse drug reactions
4. The safety profile of medicines is well characterised by the time it is marketed
5. I’m interested in reading about ADRs that are published in the medical literature
6. I’m more likely to report adverse drug reactions if:
7. There was an incentive (e.g. monetary, CPD points etc.)
8. An electronic tool was available that automatically populates ADR information from existing health datasets such as eMEDs
9. I am mandated to report and there is a consequence if I don’t
10. There is a hospital protocol mandating the reporting of adverse drug reactions
11. I see that there are other healthcare professionals reporting adverse drug reactions
12. There was a reminder alerting me to report adverse drug reactions
13. It was serious and unexpected
14. It was for a new medicine
15. It has a strong causal association with the medicine
16. There is someone monitoring our ADR reporting
17. I receive an acknowledgement from the hospital or the Australian regulator thanking me for my report
18. I don’t report adverse drug reactions because:
19. I don’t have the time
20. I fear there may be legal repercussions
21. There are no results or actions taken based on the adverse drug reactions I report
22. I forget to report at the time of the reaction
23. It was non-serious and expected
24. I usually don’t have enough information to warrant a report
25. I don’t know how to report
26. I’m uncertain of the causal relationship
27. I would rather have it published in the medical literature
28. I don’t know when I am supposed to
29. It won’t make a difference
30. My colleagues don’t
31. It would cause stress and burnout in my workload
32. I have been encouraged not to
33. What do you think can be done to increase ADR reporting in hospitals? (free text)
34. What are the most important factors that prevent you from reporting ADRs? (free text)
35. Do you have any further comments?

**Figure S1 – Behavioural Change Wheel**


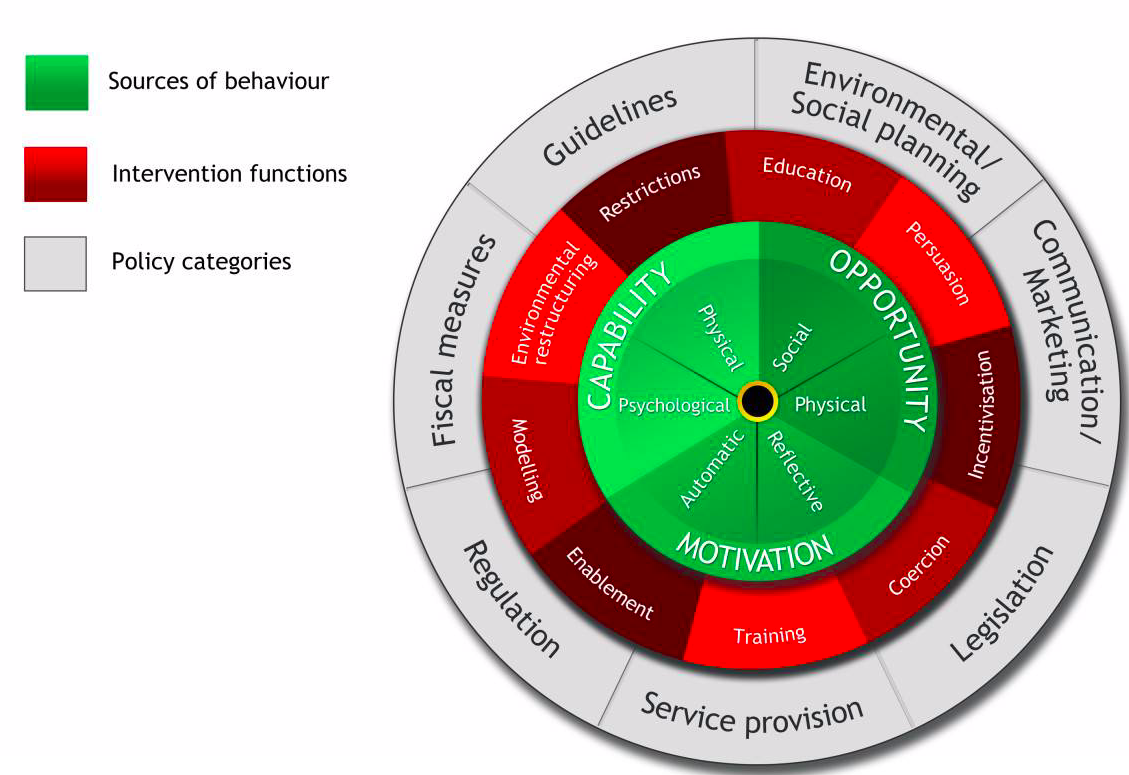

Supplement: Supplementary file 1 — Supplementary file1 (DOCX 553 KB) [file 228_2022_3326_MOESM1_ESM.docx]
